# Cardiometabolic Substrate Modification in Atrial Fibrillation: Obesity and Epicardial Adipose Tissue in the Era of Incretin-Based Therapies and Catheter Ablation

**DOI:** 10.3390/jcdd13100512

**Published:** 2026-10-10

**Authors:** Andrea Matteucci, Michela Bonanni, Luca Sgarra, Lorenzo Nesti, Marco Valerio Mariani, Nicola Pierucci, Angelica Cersosimo, Raimondo Pittorru, Federico Nardi, Furio Colivicchi

**Affiliations:** 1 Clinical and Rehabilitation Cardiology Division, San Filippo Neri Hospital, 00135 Rome, Italy; 2 Department of Cardiology, Tor Vergata University, 00133 Rome, Italy; 3 Department of Cardiovascular, Respiratory, Nephrological, Anesthesiological and Geriatric Sciences, “Sapienza” University of Rome, 00185 Rome, Italy; 4 Department of Cardiology, Regional General Hospital “F. Miulli”, 70021 Bari, Italy; lorenzo.nesti@phd.unipi.it; 5 Cardiopulmonary Laboratory, Department of Clinical and Experimental Medicine, University of Pisa, 56126 Pisa, Italy; 6 Department of Experimental and Clinical Medicine, Magna Graecia University, 88100 Catanzaro, Italy; 7 Department of Cardiac, Thoracic Vascular Sciences and Public Health, University of Padova, 35121 Padova, Italy; raimondo.pittorru@studenti.unipd.it; 8 Santo Spirito Hospital, Casale Monferrato, 15033 Alessandria, Italy

**Keywords:** atrial fibrillation, obesity, epicardial adipose tissue, atrial cardiomyopathy, GLP-1 receptor agonist, semaglutide, tirzepatide, sodium-glucose cotransporter-2, catheter ablation

## Abstract

Atrial fibrillation (AF) is increasingly understood as the electrical expression of a systemic and atrial cardiometabolic disease rather than an isolated disorder of pulmonary-vein triggers. Obesity, insulin resistance, hypertension, sleep-disordered breathing, heart failure with preserved ejection fraction, and visceral adiposity promote atrial stretch, inflammation, fibrosis, autonomic imbalance, and conduction heterogeneity. Epicardial adipose tissue (EAT), particularly the peri-left-atrial depot, may provide a local mechanistic bridge between systemic metabolic dysfunction and atrial cardiomyopathy through paracrine, inflammatory, fibrotic, and infiltrative pathways. This framework has direct therapeutic implications. Structured weight loss, cardiorespiratory fitness, alcohol reduction, and comprehensive risk-factor management can reduce AF burden and improve rhythm-control outcomes, although the magnitude of benefit varies across populations and trial designs. Incretin-based therapies have introduced a potentially important pharmacologic route to substrate modification. Semaglutide and tirzepatide produce substantial weight loss and favorable cardiovascular and heart-failure effects, while emerging observational and prospective data suggest lower AF incidence and post-ablation recurrence. However, evidence for reduced post-ablation recurrence remains predominantly non-randomized and is susceptible to residual confounding, treatment-selection effects, and healthy-user bias. Causality, weight-independent antiarrhythmic effects, optimal timing relative to ablation, and durability after treatment withdrawal remain unresolved. Observational sodium-glucose cotransporter-2 (SGLT2) inhibitor data support further investigation of cardiometabolic approaches to rhythm control, although randomized evidence has not consistently demonstrated benefit. Our review integrates mechanistic, imaging, lifestyle, pharmacologic, and electrophysiological evidence and proposes a practical model in which catheter ablation and cardiometabolic substrate modification are complementary strategies.

## 1. Introduction

Atrial fibrillation (AF) has traditionally been organized around an electrophysiological model in which triggers, most prominently from the pulmonary veins, initiate an arrhythmia that is then sustained by progressively abnormal atrial tissue. Pulmonary vein isolation (PVI) remains the procedural cornerstone of AF ablation, and contemporary trials have established the value of rhythm control and catheter ablation for symptom relief and, in selected populations, cardiovascular outcomes [1,2,3,4]. Yet the procedural view is incomplete. AF develops and recurs within a biological substrate that is continuously shaped by age, adiposity, blood pressure, glycaemic status, sleep-disordered breathing, physical fitness, alcohol exposure, heart failure, and renal dysfunction. The 2024 ESC Guidelines for the management of atrial fibrillation formalized this shift by placing comorbidity and risk-factor management at the beginning of the AF-CARE pathway, while North American guidance similarly emphasizes obesity, exercise, sleep, and alcohol as integral components of AF management [5,6].

Among modifiable drivers, obesity is especially important because it is both common and mechanistically connected to several other AF-promoting conditions. Population studies and meta-analyses demonstrate a graded relationship between body mass index (BMI) and incident AF, postoperative AF, and recurrence after ablation [7,8,9]. Obesity alters atrial loading conditions, increases left atrial size, promotes ventricular diastolic dysfunction, activates inflammatory and profibrotic pathways, changes autonomic tone, and increases visceral and epicardial fat. These processes converge on atrial cardiomyopathy, a concept that encompasses structural, architectural, contractile, and electrophysiological abnormalities capable of both maintaining AF and persisting after restoration of sinus rhythm [10,11].

This broader substrate model has become clinically actionable for two reasons. First, longitudinal risk-factor programs have shown that meaningful weight loss and improvement in cardiorespiratory fitness can reduce AF burden and improve rhythm-control durability. Second, potent anti-obesity drugs, particularly glucagon-like peptide-1 receptor agonists (GLP-1RAs) and dual glucose-dependent insulinotropic polypeptide/GLP-1 receptor agonists, can now produce weight reductions of a magnitude previously achievable mainly through bariatric surgery. At the same time, imaging can quantify epicardial adipose tissue (EAT), atrial size, strain, fibrosis, and low-voltage substrate, creating the possibility of a more granular cardiometabolic AF phenotype.

### Review Methodology

This article is a narrative review. The scope comprises obesity and weight management, epicardial adipose tissue, atrial cardiomyopathy, incretin-based therapies, SGLT2 inhibitors, and catheter ablation. PubMed/MEDLINE, Embase, and the Cochrane Library were searched from database inception through August 2026; the last searches were conducted in August 2026. Broad search concepts included atrial fibrillation and rhythm control; obesity, adiposity, weight loss, and risk-factor management; epicardial and peri-left-atrial adipose tissue; atrial cardiomyopathy and remodeling; incretin-based therapies, GLP-1 receptor agonists, semaglutide, tirzepatide, and GIP/GLP-1 agonists; SGLT2 inhibitors; and catheter ablation and post-ablation recurrence or AF burden. No language restrictions were applied. Reference lists of selected publications and articles citing them were also examined to identify additional relevant studies. These searches supported a narrative synthesis and were not conducted as a systematic-review search. Sources were selected for their clinical and mechanistic relevance to this framework and include international guidelines and consensus documents, randomized trials, prospective studies, systematic reviews and meta-analyses, and observational cohorts. Evidence is synthesized qualitatively, with attention to study design and the distinction between association and causal treatment effects; no systematic search protocol, formal risk-of-bias assessment, or quantitative evidence grading is reported.

## 2. From Obesity to Atrial Cardiomyopathy: The Cardiometabolic Substrate

### 2.1. Epidemiology and the Limitations of BMI

The epidemiological link between adiposity and AF is consistent across study designs. In the Framingham cohort, obesity independently predicted incident AF, with atrial enlargement accounting for part, but not all, of the association [7]. A large meta-analysis including more than 600,000 participants subsequently showed that each 5-unit increase in BMI was associated with excess risk across incident, postoperative, and post-ablation AF settings [8]. This relationship is clinically relevant because obesity prevalence has risen in parallel with the global AF burden [12,13]. Importantly, two individuals with the same BMI can have very different visceral adiposity, muscle mass, insulin resistance, and EAT distribution. Waist circumference, visceral fat, cardiorespiratory fitness, and organ-specific adipose depots may therefore better describe the arrhythmogenic phenotype than BMI alone.

The cardiometabolic substrate is also dynamic; weight gain, metabolic deterioration, and persistent hypertension can continue to remodel the atria after an apparently successful ablation. Conversely, sustained weight reduction may partially reverse atrial disease. This bidirectionality is one reason to avoid describing obesity merely as a baseline procedural risk marker. It is better understood as a modifiable upstream disease process whose treatment may change the natural history of AF [14].

An apparent “obesity paradox” has been reported in patients with established AF and in heart-failure populations, with some cohorts showing lower mortality or thromboembolic risk in overweight or obese categories than in normal-weight groups [15,16]. Reverse causation, residual confounding, collider or selection bias, unintentional weight loss, and the inability of BMI to distinguish adipose from lean mass can all contribute to this pattern. Importantly, these prognostic associations in prevalent disease differ conceptually from studies evaluating intentional, sustained weight reduction as a strategy to modify AF substrate and recurrence.

### 2.2. Hemodynamic, Structural, and Electrical Remodeling

Obesity imposes several mechanical stresses on the atria. Increased circulating volume, higher ventricular filling pressures, hypertension, and sleep-disordered breathing increase atrial wall stress and promote left atrial enlargement. Obesity-related HFpEF adds an important amplifier: elevated filling pressures, systemic inflammation, impaired ventricular-atrial coupling, and exercise intolerance can sustain an environment in which AF and heart failure reinforce one another. Experimental models support a direct causal contribution of obesity to atrial disease. Progressive weight gain in large-animal models produces atrial dilation, conduction slowing, increased fractionation, interstitial fibrosis, and greater AF inducibility [17,18]. Human electroanatomical studies similarly identify lower atrial voltage, slowed conduction, and greater substrate complexity in obesity, particularly adjacent to epicardial fat [19].

**Figure 1:**
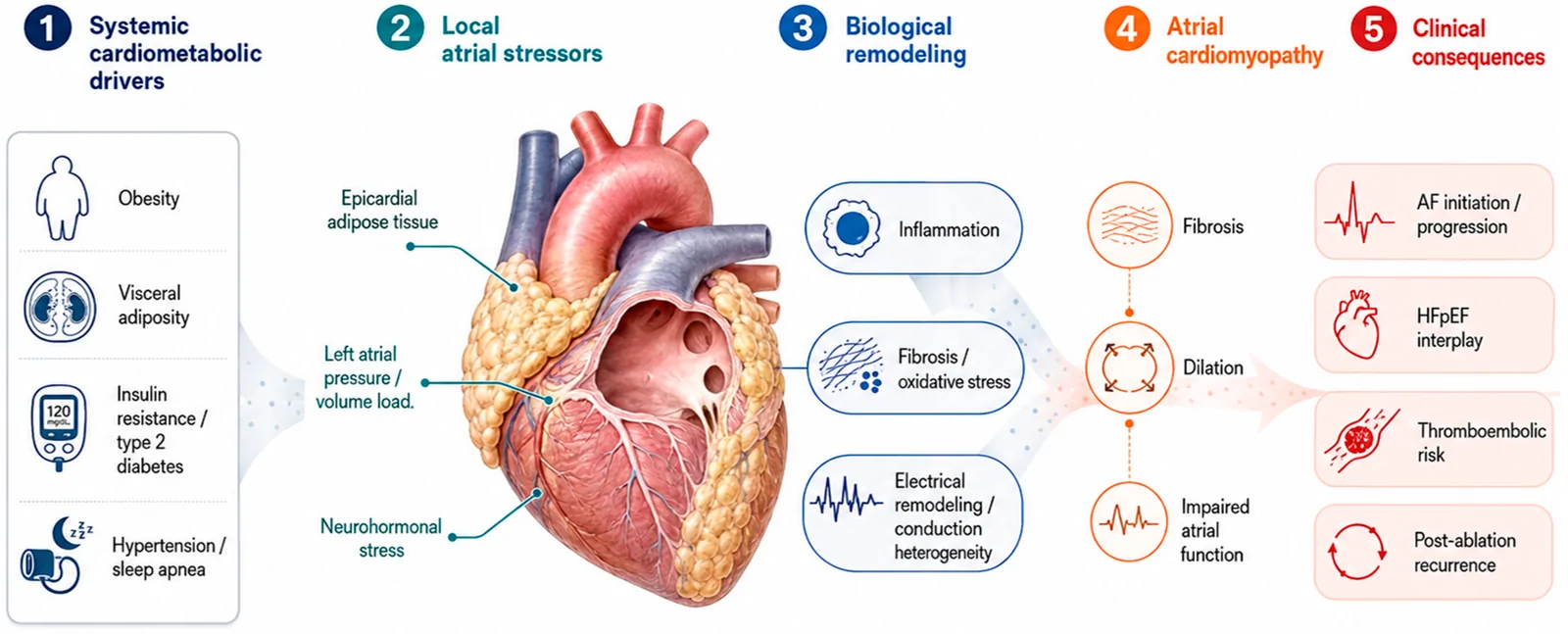
Proposed pathophysiological continuum linking cardiometabolic disease to atrial fibrillation. The schematic summarizes the proposed sequence from systemic cardiometabolic drivers (obesity, visceral adiposity, insulin resistance/type 2 diabetes, and hypertension/sleep apnea), through local atrial stressors including epicardial adipose tissue, increased left-atrial pressure/volume load, and neurohumoral stress, to inflammatory, fibrotic, and electrical remodeling. These processes converge on atrial cardiomyopathy and its clinical consequences, including AF initiation/progression, HFpEF interplay, thromboembolic risk, and post-ablation recurrence. Abbreviations: AF, atrial fibrillation; HFpEF, heart failure with preserved ejection fraction.

Metabolic overload activates oxidative stress, altered calcium handling, transforming growth factor-beta signaling, fibroblast activation, and inflammatory pathways. NLRP3 inflammasome signaling is of particular interest because it links metabolic stress to both triggered activity and re-entry. Cardiomyocyte-specific NLRP3 activation can produce atrial ectopy and electrical remodeling, while obesity-associated inflammasome activation promotes atrial fibrosis and arrhythmia susceptibility [20,21]. These mechanisms provide biological plausibility for a treatment strategy that aims not only to reduce body weight but also to attenuate inflammatory and profibrotic signaling; inflammation has long been recognized as a central component of AF initiation and maintenance [22] (Figure 1).

### 2.3. The Obesity-AF-HFpEF Continuum

AF, obesity, and HFpEF share increased visceral adiposity, impaired natriuretic peptide biology, inflammation, endothelial dysfunction, renal sodium retention, reduced exercise capacity, and elevated cardiac filling pressures. Once AF develops, loss of coordinated atrial contraction and rapid irregular ventricular activation can further worsen functional capacity and filling pressures. Conversely, congestion and elevated left atrial pressure can accelerate atrial remodeling. This reciprocal relationship helps explain why interventions that improve the obesity-related HFpEF phenotype may plausibly influence AF even when they are not classical antiarrhythmic drugs. A reduction in post-ablation AF recurrence after a metabolic intervention could reflect several mechanisms: less atrial stretch, reduced EAT, improved blood pressure, better sleep apnea control, lower inflammatory activity, improved ventricular diastolic function, or direct electrophysiological effects.

## 3. Epicardial Adipose Tissue: Local Effector, Biomarker, and Potential Target

### 3.1. Why EAT Is Biologically Different from Generalized Obesity

Unlike subcutaneous fat, EAT has no fascial boundary separating it from the underlying myocardium, shares the coronary microcirculation, and is anatomically concentrated around the atrioventricular grooves, coronary arteries, and posterior left atrium. In health, EAT has metabolic, mechanical, and thermogenic functions; in cardiometabolic disease, it can expand and acquire a proinflammatory, profibrotic phenotype [23,24]. The EAT secretome can induce atrial myocardial fibrosis, with mediators such as activin A providing a mechanistic link between adipose dysfunction and atrial structural remodeling [25].

The atrial consequences are likely both paracrine and anatomical. EAT-derived cytokines and adipokines may diffuse into adjacent myocardium; fatty infiltration may separate myocardial bundles and increase conduction discontinuity; oxidative and inflammatory signaling may alter ion-channel function; and the close relationship between EAT and autonomic ganglionated plexi may modulate sympathovagal activity. The peri-left-atrial EAT may be more relevant to AF than total cardiac fat because it is in direct contact with the posterior atrial wall and pulmonary-vein antra, regions central to AF initiation and ablation [26,27].

### 3.2. EAT and AF Severity, Substrate, and Recurrence

Clinical imaging studies consistently associate higher pericardial or epicardial fat burden with AF presence and severity. Wong et al. reported that pericardial fat was associated with AF severity and ablation outcome independently of conventional measures of adiposity [28]. Subsequent computed tomography (CT) studies linked larger EAT volumes, including left-atrial-specific EAT, to recurrence after catheter ablation [29,30]. Meta-analyses have reinforced the signal, although effect sizes and heterogeneity vary according to the method used to quantify fat, the AF phenotype, imaging protocol, and outcome ascertainment [31,32].

Greater left-atrial EAT has been associated with low-voltage regions, suggesting a relationship between adipose proximity and atrial fibrosis or myocyte loss [33]. Studies combining EAT measures with left atrial low-voltage area have reported improved prediction of recurrence compared with either marker alone [34].

### 3.3. EAT Study: Attenuation, Radiomics, and Automated Quantification

CT attenuation may reflect adipocyte composition and inflammatory state, whereas radiomics can characterize spatial heterogeneity, texture, and distribution that are invisible to conventional measurements. Early studies suggest that peri-left-atrial EAT radiomic signatures can add prognostic information for post-ablation recurrence [35,36]. Automated segmentation using deep-learning workflows substantially reduces the manual burden of EAT quantification and has shown feasibility for recurrence prediction [37].

Cardiac magnetic resonance (CMR) provides complementary information by characterizing atrial fibrosis rather than adipose tissue alone. The DECAAF study demonstrated a relationship between late gadolinium enhancement-defined atrial fibrosis and ablation outcome, while DECAAF II did not show that empirically adding MRI-guided fibrosis ablation to PVI improved recurrence in persistent AF [38,39]. Recent multimodality consensus documents emphasize integrated assessment of atrial anatomy and function [40], and dedicated reviews have highlighted the evolving role of CMR in defining atrial cardiomyopathy and guiding substrate-oriented strategies [41] (Table 1).

Continuous rhythm monitoring provides a dynamic AF-burden phenotype that can be more informative than a binary recurrence endpoint [42]. AI-enabled ECG analysis has identified patients with AF during sinus rhythm [43], while broader electrophysiology applications include approaches integrating ECG, imaging, and clinical data [44]. Their use for individualized recurrence prediction still requires calibration and prospective external validation.

## 4. Substrate Modification Before and After Ablation

### 4.1. Structured Weight Loss and Comprehensive Risk-Factor Management

In a randomized trial, structured weight reduction combined with cardiometabolic risk-factor management reduced AF symptoms and burden and favorably affected cardiac remodeling [45]. The ARREST-AF cohort then showed that aggressive management of weight, blood pressure, glycaemia, lipids, sleep apnea, smoking, and alcohol was associated with substantially better long-term rhythm outcomes after ablation [46]. In LEGACY, sustained weight loss of at least 10% was associated with markedly greater arrhythmia-free survival, whereas weight fluctuation attenuated benefit [47]. CARDIO-FIT added that cardiorespiratory fitness predicted rhythm outcome, and improvement in fitness amplified the benefit associated with weight loss [48].

In REVERSE-AF, greater sustained weight loss and risk-factor control were associated with regression toward less persistent AF phenotypes [49]. However, the evidence is not uniformly positive. SORT-AF confirmed the feasibility and safety of supervised weight reduction around ablation but did not reproduce the magnitude of rhythm benefit seen in earlier cohort-based programs [50]. Meta-analytic evidence supports an inverse dose-response relationship between weight loss and recurrence, but heterogeneity remains substantial and observational designs can be strongly influenced by adherence, health behavior, and selection bias [51]. These same concerns apply to lifestyle-clinic cohorts: patients who achieve and maintain substantial weight loss may be more motivated, adherent, and engaged with other health-promoting behaviors, which can independently improve rhythm outcomes and complicate comparisons with pharmacological strategies.

The recent randomized ARREST-AF trial is therefore particularly important because it strengthens the causal case for structured risk-factor and weight management as an adjunct to ablation [52]. Delaying effective rhythm control for prolonged or unsuccessful weight loss could allow further atrial remodeling.

### 4.2. Exercise, Alcohol, Sleep Apnea, and Weight Stability

Exercise improves fitness, blood pressure, insulin sensitivity, and functional status, and its benefit may not be captured by the scale. CARDIO-FIT suggests that improving objective exercise capacity is associated with better arrhythmia control [48]. Alcohol is another modifiable exposure with randomized evidence: abstinence in regular drinkers with AF reduced recurrence and AF burden [53]. Obstructive sleep apnea is highly prevalent in obesity and AF, and observational and meta-analytic studies associate untreated sleep apnea with recurrence after ablation and CPAP treatment with lower recurrence, although randomized evidence remains less definitive [54,55]. The LEGACY experience showed that substantial weight fluctuation can erode the rhythm benefit of weight loss [47]. This has direct relevance to incretin therapy because discontinuation commonly leads to weight regain. An intervention that produces rapid pre-ablation weight loss but is stopped soon after the procedure may not provide durable substrate modification.

**Figure 2:**
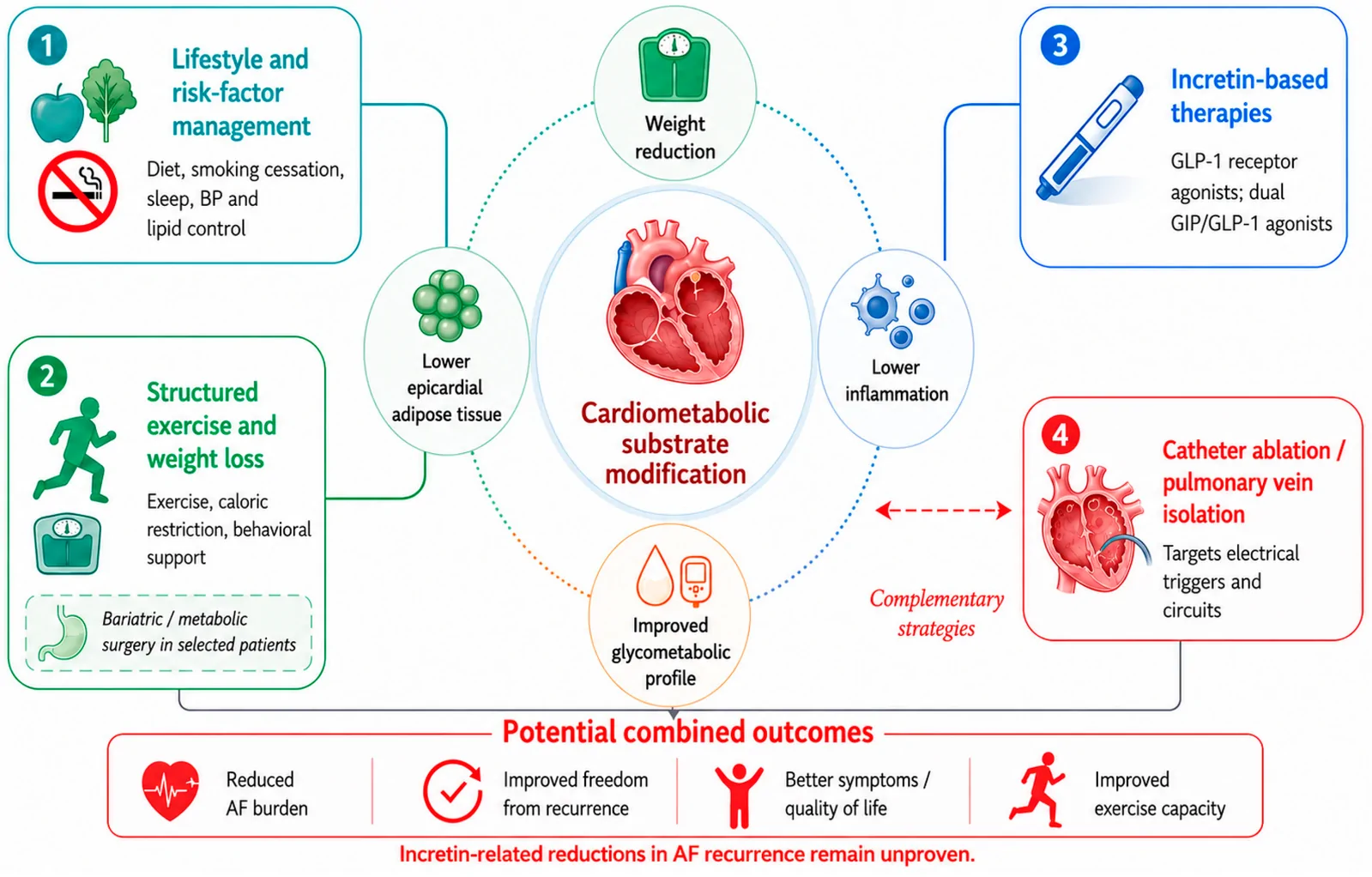
Therapeutic model of cardiometabolic substrate modification. Lifestyle and comprehensive risk-factor management, structured exercise and weight loss (including bariatric/metabolic surgery in selected patients), and incretin-based therapy act on complementary components of the cardiometabolic substrate, including body weight, epicardial adiposity, inflammation, and glycometabolic status. Catheter ablation targets electrical triggers and circuits in parallel. The potential combined clinical outcomes are lower AF burden, greater freedom from recurrence, improved symptoms/quality of life, and better exercise capacity; the pharmacological contribution to rhythm control remains under investigation. Incretin-related reductions in AF recurrence remain unproven. Abbreviations: AF, atrial fibrillation; BP, blood pressure; GLP-1, glucagon-like peptide-1; GIP, glucose-dependent insulinotropic polypeptide.

Bariatric surgery offers a useful biological experiment because it can generate large, durable weight loss and broad metabolic improvement. In the Swedish Obese Subjects cohort, bariatric surgery was associated with lower risk of new-onset AF [56]. In morbidly obese patients referred for AF ablation, observational studies reported lower recurrence among those who had undergone bariatric surgery before ablation and outcomes approaching those of non-obese comparators [57,58]. In the modern era, potent incretin-based therapy creates the possibility of achieving substantial weight loss without surgery in a much larger proportion of patients (Figure 2).

## 5. Incretin-Based Therapies: A New Pharmacologic Route to Substrate Modification

### 5.1. Why GLP-1/GIP Signaling Is Relevant to AF

GLP-1RAs were developed for glycaemic control, but they have also become relevant in AF. Semaglutide can produce double-digit percentage weight loss in obesity, and tirzepatide can achieve even greater reductions in many patients [59,60,61]. In addition to weight loss, these agents improve glycaemia, blood pressure, inflammatory markers, sleep apnea severity in some populations, and heart-failure symptoms; they may also influence visceral and para-cardiac fat.

Three non-mutually exclusive mechanisms may account for their potential relevance to AF. The first is a weight-mediated effect: less visceral and cardiac adiposity reduces atrial loading and inflammatory signaling. The second is an indirect cardiometabolic effect: improved blood pressure, glycaemia, kidney-heart interactions, sleep-disordered breathing, and HFpEF physiology reduce atrial stress even beyond the contribution of body weight. The third is a direct cardiac or adipose-tissue effect through GLP-1-related signaling. Human EAT expresses incretin-related receptors and is metabolically responsive, but the clinical importance of direct atrial electrophysiological effects remains uncertain [62].

### 5.2. Cardiovascular and HFpEF Trials Provide the Biological Context

Large outcome trials have established that incretin-based therapy can modify cardiovascular disease at scale. SELECT showed that semaglutide reduced major adverse cardiovascular events in patients with overweight or obesity and established cardiovascular disease without diabetes [63]. In STEP-HFpEF, semaglutide improved symptoms, physical limitations, exercise capacity, and weight in obesity-related HFpEF; similar benefits were observed in patients with concomitant type 2 diabetes, and pooled analyses strengthened the consistency of the phenotype-specific effect [64,65,66]. Because AF is common in HFpEF, the prespecified STEP-HFpEF program analysis showing benefit across patients with and without AF is particularly relevant to the substrate model [67].

In the SUMMIT trial, tirzepatide reduced the composite of cardiovascular death or worsening heart failure in patients with obesity-related HFpEF and improved health status [68]. A CMR substudy documented reductions in left ventricular mass and para-cardiac adipose tissue, illustrating that pharmacologic weight loss is accompanied by measurable cardiac structural changes [69]. These studies were not designed to test AF ablation outcomes, but they establish that incretin-based treatment can modify the exact systemic phenotype in which AF often develops and recurs.

Meta-analyses of randomized semaglutide trials suggest that incident AF may be reduced compared with control treatment [70,71,72]. However, AF was generally not a primary adjudicated endpoint, monitoring intensity varied, and study populations were selected for diabetes, obesity, or cardiovascular risk rather than AF susceptibility. A lower rate of clinically detected AF is therefore hypothesis-generating rather than definitive proof of a direct antiarrhythmic effect.

### 5.3. GLP-1RAs After Catheter Ablation: Emerging Evidence

In a large database analysis, preprocedural GLP-1RA use was not associated with lower 12-month AF recurrence, whereas a subsequent meta-analysis of propensity-matched observational studies reported an overall reduction in recurrence [73,74]. A 2025 study of obese patients found a lower long-term recurrence rate among GLP-1RA-treated patients after ablation [75]. In a prospective, preference-based, non-randomized cohort of 437 patients, all with type 2 diabetes and BMI ≥24 kg/m^2^, semaglutide treatment was associated with greater weight loss and a lower risk of atrial arrhythmia recurrence [76]. In predominantly non-diabetic patients with obesity-related paroxysmal AF, semaglutide used as adjunctive therapy to ablation was also associated with improved rhythm outcomes [77]. These non-randomized signals remain susceptible to confounding by indication, differential adherence and access to care, and healthy-user bias.

The randomized LEAF study added that three months of liraglutide before ablation did not produce a greater reduction in left-atrial epicardial adipose tissue than structured risk-factor modification alone, so its prespecified mechanistic primary endpoint was neutral. Nevertheless, freedom from AF or atrial flutter at 1 year was higher in the liraglutide group, a secondary finding that raises the possibility of effects not fully mediated by short-term change in weight or EAT volume but requires confirmation in larger trials [78]. In a retrospective, propensity-matched cohort of non-obese patients, GLP-1RA use was associated with less cardioversion and antiarrhythmic-drug use, fewer heart-failure exacerbations, and fewer hospitalizations at 1 year [79]. These findings concern arrhythmia-related interventions and selected clinical outcomes rather than directly monitored AF recurrence; they do not establish a weight-independent antiarrhythmic effect, and residual confounding remains difficult to exclude. The most recent meta-analysis integrating GLP-1 agonists with other weight-loss strategies found that weight-loss interventions overall reduced recurrence and identified a dose-response relationship between the magnitude of weight reduction and arrhythmia outcome; importantly, sustained continuation after ablation appeared to matter [80].

It is plausible that incretin-based therapies improve the durability of AF ablation in selected patients with obesity, especially when treatment produces substantial and sustained weight loss (Table 2).

### 5.4. Safety Considerations Relevant to Rhythm-Control Populations

Potential arrhythmic and functional trade-offs also deserve explicit consideration. GLP-1 receptor agonists and related incretin-based therapies produce a modest but consistent increase in resting heart rate, with the magnitude varying by agent and dose [81]. Although meta-analyses of randomized trials have not demonstrated an overall increase in AF, drug- and dose-specific signals and uncertainty around ventricular arrhythmias mean that heart-rate effects should not be assumed to be electrophysiologically neutral in every patient [81,82]. In addition, rapid pharmacological weight loss includes some loss of lean mass; in the STEP 1 body-composition substudy, semaglutide reduced total lean mass even though fat mass fell proportionally more and the lean-to-fat mass ratio improved [83]. In older or frail patients with AF, preservation of muscle mass and physical function should therefore be considered alongside the metabolic benefits of intensive weight loss.

**Table 2:** Selected clinical studies of cardiometabolic substrate modification and AF outcomes.

Study	Design/Population (*n*)	Intervention or Exposure	Primary Endpoint/Principal AF Finding	Key Interpretive Limitation
Abed et al. 2013 [45]	Randomized trial; overweight/obese symptomatic AF (*n* = 150)	Structured weight loss + risk-factor management	AF symptom burden/severity: reduced with structured weight loss	Relatively small trial; bundled intervention
ARREST-AF cohort 2014 [46]	Prospective cohort; AF ablation + risk factors (*n* = 149)	Aggressive multi-risk-factor program	Post-ablation arrhythmia-free survival: better with RFM	Non-randomized; adherence/selection bias
LEGACY 2015 [47]	Longitudinal cohort; BMI ≥ 27 kg/m^2^ (*n* = 355)	Goal-directed weight management	AF burden/freedom: greatest with ≥10% sustained weight loss	Observational; highly motivated cohort
CARDIO-FIT 2015 [48]	Cohort; obese symptomatic AF (*n* = 308)	Fitness level and fitness gain	Rhythm outcome: higher/gaining fitness associated with lower recurrence	Exercise capacity may mark overall health behavior
REVERSE-AF 2018 [49]	Longitudinal cohort (*n* = 355)	Weight/risk-factor change	AF phenotype: greater loss associated with regression toward less persistent AF	Post hoc observational framework
SORT-AF 2021 [50]	Randomized trial around AF ablation (*n* = 133)	Supervised obesity reduction	AF burden 3–12 months: no significant overall between-group difference	Smaller between-group weight difference
Bariatric surgery 2019 [57]	Retrospective morbid-obesity ablation cohort (*n* = 239)	Pre-ablation bariatric surgery	AF recurrence: lower after pre-ablation bariatric surgery	Selection and referral bias
ARREST-AF RCT 2025 [52]	Randomized trial; AF ablation + elevated BMI/risk factors (*n* = 122)	Structured risk-factor and weight management	12-month freedom from AF: higher with structured LRFM	Open-label, multifactorial intervention
GLP-1RA post-ablation cohorts [73,75]	Propensity-matched database cohorts (*n* = 3250 and *n* = 3116)	GLP-1 receptor agonist use	Composite recurrence proxies: neutral in [73], lower in obese patients in [75]	Confounding by indication and healthy-user bias
Semaglutide prospective study 2025 [76]	Prospective, preference-based, non-randomized AF ablation cohort; all participants had type 2 diabetes and BMI ≥ 24 kg/m^2^ (*n* = 437)	Semaglutide	12-month freedom from atrial arrhythmia: higher with semaglutide	Non-randomized treatment allocation; residual confounding
Semaglutide + ablation 2026 [77]	Propensity-matched obesity-related paroxysmal AF cohort (*n* = 362)	Adjunctive semaglutide	Atrial tachyarrhythmia recurrence: lower with semaglutide	Non-randomized; requires randomized confirmation
LEAF 2026 [78]	Randomized open-label; overweight/obese AF (*n* = 59)	Liraglutide + risk-factor management	LAEAT change: neutral primary endpoint; AF/AFL freedom higher (secondary)	Small trial; rhythm outcome secondary
GLP-1RA in non-obese patients 2026 [79]	Retrospective, propensity-matched AF ablation cohort; BMI ≤ 29.9 kg/m^2^ (*n* = 3498; 1749 per group)	GLP-1 receptor agonist use	Less cardioversion and antiarrhythmic-drug use, fewer heart-failure exacerbations and hospitalizations at 1 year; repeat ablation, stroke and mortality similar	Administrative-data outcomes, rather than directly monitored AF recurrence; residual confounding
Weight-loss meta-analysis 2026 [80]	2 RCTs + 11 cohorts (*n* = 5415)	GLP-1RA, lifestyle/RFM, bariatric surgery	AF recurrence: lower overall; dose-response with % weight loss	Heterogeneous interventions; mostly non-randomized evidence
SGLT2i evidence [84,85,86,87]	Observational cohorts/meta-analyses + DARE-AF RCT (DARE-AF *n* = 200)	SGLT2 inhibitor use	Observational recurrence signal; DARE-AF primary endpoint of AF burden at 3 months neutral	Discordance between observational and randomized evidence

Abbreviations: AF, atrial fibrillation; AFL, atrial flutter; BMI, body mass index; GLP-1, glucagon-like peptide-1; GLP-1RA, GLP-1 receptor agonist; LAEAT, left atrial epicardial adipose tissue; LRFM, lifestyle and risk-factor management; RCT, randomized controlled trial; RFM, risk-factor modification; SGLT2i, sodium-glucose cotransporter-2 inhibitor.

## 6. SGLT2 Inhibitors and the Broader Concept of Cardiometabolic Antiarrhythmic Therapy

Sodium-glucose cotransporter-2 (SGLT2) inhibitors have established heart-failure and renal benefits and also exert favorable effects on natriuresis, filling pressures, metabolic efficiency, body weight, blood pressure, inflammation, and potentially atrial remodeling. Several observational analyses in patients with diabetes undergoing AF ablation have associated SGLT2 inhibitor use with lower recurrence [84], and subsequent meta-analyses reported broadly consistent associations [85,86]. However, the randomized DARE-AF trial provided an important counterpoint: three months of dapagliflozin after ablation did not reduce AF burden at three months, the primary endpoint, in patients with persistent AF without established indications for an SGLT2 inhibitor; AF recurrence, a secondary endpoint, was also not reduced [87]. The result weakens any assumption of a universal class-level antiarrhythmic effect and highlights the importance of treatment indication, phenotype, timing, duration, and endpoint selection.

Observational signals across pharmacologic classes suggest that modification of hemodynamic and cardiometabolic physiology may influence rhythm outcomes, but DARE-AF shows that this cannot be inferred from cardiometabolic benefit alone.

## 7. Catheter Ablation in a Metabolically Active Substrate

The discovery of pulmonary-vein triggers transformed AF treatment [1], and modern ablation technologies continue to improve procedural efficiency and safety [88,89,90]. Long-term ablation meta-analysis data show that repeat procedures are often needed, particularly as substrate complexity increases [91]. Continuous-monitoring trials such as CIRCA-DOSE also illustrate why AF burden and episode duration can provide a richer assessment of rhythm-control efficacy than a single binary recurrence endpoint [92].

Multicenter studies have associated higher BMI with reduced ablation efficacy in some populations even after adjustment for conventional clinical factors [93,94]. This can occur despite technically successful PVI when the atrium remains exposed to elevated filling pressures, EAT-derived inflammatory signaling, fibrosis, sleep apnea, and metabolic dysfunction.

The introduction of pulsed field ablation (PFA) has rapidly changed the procedural landscape by creating non-thermal lesions with relative tissue selectivity. Early studies and the ADVENT randomized trial established effective PVI with a distinct safety profile compared with thermal ablation [95,96]. Contemporary consensus recognizes PFA as an important ablation modality [97]. In obese patients, early comparative data suggest that PFA can be performed effectively and may offer procedural advantages, but obesity-related substrate remains relevant regardless of energy source [98]. Energy technology and substrate biology operate on different levels: one determines how reliably the lesion set is created; the other influences whether the atrium remains arrhythmogenic after the lesion set is complete. Ablation can substantially improve symptoms and quality of life in obese patients, as demonstrated in CABANA [99], but symptom improvement is not perfectly aligned with elimination of all AF episodes. Comparative real-world data suggest that perceived symptom and quality-of-life gains can be achieved across different energy sources and BMI [100]. Conversely, patients may continue to report fatigue, anxiety, exercise limitation, or treatment burden even after a procedure judged technically successful, highlighting a quality-of-life gap that rhythm endpoints alone do not capture [101].

Substantial pharmacologically induced weight loss can change body weight and composition over months, supporting periodic reassessment of weight, renal function, adherence, and interacting medications. Long-term anticoagulation remains guided by thromboembolic risk rather than apparent ablation success [5,6]. Recent meta-analyses of anticoagulant discontinuation after ablation inform this discussion but do not establish that weight loss itself permits withdrawal [102,103].

## 8. A Practical Clinical Framework

A pragmatic pathway can be organized around four phases. First, at AF diagnosis, clinicians should identify modifiable substrate drivers rather than record BMI alone: weight trajectory, waist circumference, blood pressure, diabetes or insulin resistance, sleep apnea, alcohol exposure, physical activity, HFpEF features, renal function, and relevant medications. Second, cardiometabolic treatment should start early and in parallel with rhythm control. In patients with obesity, a clinically meaningful weight-loss target should be individualized; structured lifestyle therapy remains foundational, while GLP-1RA or dual GIP/GLP-1 therapy can be considered when indicated by obesity, diabetes, or cardiovascular guidelines, while their potential antiarrhythmic effects remain under investigation [104]. Third, ablation should not be unnecessarily postponed when symptoms, AF progression, or clinical context favor intervention. Fourth, substrate treatment should continue after ablation, because recurrence risk reflects the future trajectory of the atrium as much as its condition on procedure day.

The intensity of follow-up should reflect phenotype. A patient with severe obesity, large peri-left-atrial EAT, HFpEF, sleep apnea, and persistent AF has a different biological recurrence risk from a lean patient with paroxysmal trigger-driven AF. In the former, multidisciplinary obesity care, pharmacologic weight management, sleep therapy, blood-pressure control, exercise rehabilitation, and serial rhythm assessment are not optional add-ons; they are part of the rhythm-control strategy. Conversely, escalating metabolic therapy in a metabolically healthy patient solely because an AF ablation is planned is not supported by current evidence.

## 9. Research Agenda: What Must Be Proven Next?

The priority is a randomized trial specifically designed around AF ablation and obesity. Patients should be randomized to guideline-directed risk-factor management with or without a potent incretin-based therapy, ideally initiated sufficiently before ablation to document early metabolic response and continued long enough afterward to assess durability. Continuous or high-intensity rhythm monitoring should quantify AF burden rather than relying only on symptom-triggered ECGs. Stratification should include AF type, diabetes status, sex, baseline BMI or waist circumference, HFpEF phenotype, and EAT burden. The primary analysis should test rhythm outcomes, while mechanistic substudies should measure EAT volume/attenuation, left atrial size and strain, CMR fibrosis where feasible, inflammatory biomarkers, and changes in exercise capacity.

Several questions should be pre-specified. Is benefit proportional to weight loss, or is there residual treatment effect after accounting for achieved weight change? Does starting treatment before ablation confer greater benefit than starting immediately afterward? Is continuous therapy required, and what happens after discontinuation or weight regain? Do semaglutide and tirzepatide differ in AF effect at comparable weight loss? Are patients without diabetes but with high EAT particularly responsive? Can SGLT2 inhibition and incretin therapy provide additive benefit in HFpEF or diabetes? Given the economic burden of AF and the potential effect of ablation on healthcare utilization [105], does substrate modification reduce repeat ablation and health-care utilization enough to offset drug costs? Current observational datasets cannot answer these questions robustly because treatment selection, adherence, socioeconomic status, and health behavior are deeply intertwined with exposure.

## 10. Limitations

This review has limitations. It is a narrative, non-systematic synthesis and therefore remains susceptible to selection and publication bias because source selection is qualitative rather than systematic. The evidence linking incretin therapy to AF recurrence after ablation is still dominated by retrospective or non-randomized cohorts, and, as of the August 2026 literature search, published, adequately powered randomized evidence establishing the efficacy of incretin therapy in reducing post-ablation AF recurrence or AF burden was lacking. LEAF was a small randomized study with a mechanistic primary endpoint (change in left-atrial EAT volume); its favorable secondary rhythm finding requires confirmation in larger trials [78]. EAT studies also use heterogeneous definitions, anatomical boundaries, CT attenuation thresholds, segmentation methods, and imaging platforms, limiting direct comparability and preventing a universally accepted clinical threshold. Finally, the field is evolving rapidly, so conclusions regarding newer pharmacological and imaging strategies should be considered provisional pending dedicated randomized and externally validated studies.

## 11. Conclusions

Obesity and related cardiometabolic disorders promote a dynamic atrial substrate through hemodynamic stress, systemic inflammation, fibrosis, autonomic remodeling, and expansion or dysfunction of EAT. The evidence, however, has not reached the point at which incretin therapy can be considered an established antiarrhythmic adjunct independent of its approved cardiometabolic indications. Most post-ablation recurrence data for incretin therapy remain observational and vulnerable to confounding by indication, differential adherence, and healthy-user effects; negative or neutral findings remind us that agent, population, treatment duration, adherence, and achieved weight loss matter. SGLT2 inhibitor data reinforce the broader hypothesis that modifying cardiometabolic physiology can influence AF, but randomized AF-specific trials are still needed.

## Figures and Tables

**Table 1:** Candidate markers for cardiometabolic atrial substrate phenotyping.

Marker/Modality	Biological Signal	Potential Clinical Use	Current Limitation
BMI and waist circumference	Global and central adiposity	Screening; longitudinal weight target	Limited discrimination of visceral and cardiac fat
Cardiorespiratory fitness	Integrated functional reserve	Exercise prescription; prognosis	Requires standardized testing; affected by comorbidity
Total EAT volume on CT/CMR	Cardiac visceral adiposity	Risk enrichment; metabolic phenotype	No universally accepted threshold
Peri-left-atrial EAT volume	Local adipose-myocardial interaction	Potential prediction of recurrence [29,30,32]	Segmentation methods vary
EAT attenuation/radiomics	Adipose composition and heterogeneity	Emerging prediction of recurrence [36]	Limited external validation and harmonization
LA LGE-CMR fibrosis	Structural atrial cardiomyopathy	Prognosis and research phenotyping [38,41]	Acquisition/segmentation complexity; treatment implications uncertain
LA volume and strain	Chamber remodeling and mechanical function	Routine phenotyping; serial response	Load-dependent; vendor variability
Electroanatomical low-voltage area	Electrical/structural substrate	Invasive substrate definition [34]	Mapping-dependent; not available before procedure
AF burden from continuous monitoring [42]	Dynamic arrhythmia phenotype	Better outcome measure than binary recurrence	Device availability; clinically meaningful thresholds unresolved
AI-based approaches [43,44]	Integrated ECG, imaging and clinical phenotype	Future individualized recurrence prediction	Bias, calibration, explainability, and prospective validation

Abbreviations: AF, atrial fibrillation; AI, artificial intelligence; BMI, body mass index; CMR, cardiac magnetic resonance; CT, computed tomography; EAT, epicardial adipose tissue; ECG, electrocardiogram; LA, left atrial; LGE, late gadolinium enhancement.

## Data Availability

No new data were created or analyzed in this study. Data sharing is not applicable to this article.
